# Explaining and responding to the Ebola epidemic

**DOI:** 10.1186/s13010-015-0027-8

**Published:** 2015-03-04

**Authors:** Solomon Benatar

**Affiliations:** Bioethics Centre, Philosophy Department, Upper Campus, University of Cape Town, 3.03 Humanities Building, University Ave, Rondebosch, Cape Town South Africa

**Keywords:** Africa, Ebola, Causal explanations, Public health, Social development, Tipping point, Ethics, International relations, Global political economy, Human rights, Climate change, Progress

## Abstract

The Ebola epidemic in West Africa is not merely a biomedical problem that can be seen in isolation and dealt with only through emergency medical rescue processes. The ethical dilemmas surfaced by this epidemic are also not confined to the usual micro-ethical problems associated with medical care and medical research. The pandemic, as one of many manifestations of failed human and social development that has brought the world to dangerous ‘tipping points’, requires deep introspection and action to address upstream causal processes.

The Ebola epidemic in West Africa is not merely a biomedical problem that can be seen in isolation and dealt with only through emergency medical rescue processes. The ethical dilemmas surfaced by this epidemic are also not confined to the usual micro-ethical problems associated with medical care and medical research.

The most superficial explanation for the Ebola epidemic in West Africa is that it is merely a biomedical problem arising from transmission of an animal virus to humans. A deeper explanation relates to failure to install well-known public health measures necessary to prevent the rapid spread of infection, and failure to develop vaccines and drugs for use in good time [1]. These are notable failures, given several small epidemics since the emergence of Ebola in 1976 and knowledge of the rapidly fatal outcome of this infection.

The deepest, less easily perceived and largely ignored explanation is that the epidemic is one of many manifestations of failed human and social development that has brought some countries (for example Liberia [2]), Sierra Leone and the world [3-5] to dangerous ‘tipping points ’ ’as exemplified by:

- Widening disparities in wealth and health with extensive severe poverty
- Resurgence and global spread of old infectious diseases – tuberculosis, malaria
- Rising drug resistance
- Emergence of new infectious diseases: HIV, SARS, Ebola etc.
- Reduction in energy, water and food security
- Failure to achieve human rights and human potential more extensively
- Major economic crises, corruption, exploitation and unrepayable debt
- Escalating social conflict, proliferation of fundamentalist groups, ethnic and religious conflict, anarchy and displacement of millions of people
- Failure to reproduce caring social institutions such as educational, health and other infra-structural services
- Ongoing population growth
- Wasteful consumption patterns by the privileged
- Global warming and environmental degradation

Upstream causal forces that include the structure of the global political economy, corruption, the distorted application of values such as freedom and human rights, demographic changes resulting from wars, refugees, an ageing population and lack of visionary local and global leadership, are central to creating and perpetuating an unstable, unsustainable trajectory of human ‘progress’ towards what has been labeled a ‘global organic crisis.’ This comprises many overlapping and interlinked components within systematically entrenched forms of local and global structural social violence [2,6-8].

The ethical dilemmas surfaced by this epidemic are similarly not confined to the usual micro-ethical problems associated with medical care and medical research. The epidemic raises the necessity of extending the bioethics discourse beyond interpersonal ethics to the ethics of public health and the ethics of the international relations that play such a central role in shaping the health of whole populations [5,6].

Mobilizing clinical support to treat victims is the obvious urgent and necessary response, together with a range of emergency measures, including burying the infected dead, which could limit ongoing spread of the disease to local and regional populations. Because of severe poverty and weak local health services in the endemic areas, external humanitarian organizations like Médecins Sans Frontières (MSF) play a vital role in responding, and philanthropic support for its work is essential. MSF was almost alone in its initial endeavours although international support was subsequently forthcoming. However, MSF and its courageous members cannot be responsible for building the required public health infrastructure, and the World Health Organization has neither been an effective agent for such a task specifically, nor for improving global health generally.

## Ebola as a symptom of a global organic crisis

It has become essential to acknowledge the socio-political-ideological forces that have generated local crises such as in Liberia [2] and Sierra Leone, the linkages of these crises to the ‘global organic crisis,’ and for serious attempts to be made to address these more comprehensively through changes in attitudes and responses to the health of whole populations at this critical time in human history [9]. The barrier to meaningful change lies in failing to admit that our predicament is at least in part attributable to privileged populations wastefully consuming highly disproportionate levels of energy, to their having inordinate expectations of what life owes them and to imagining that ‘business as usual’ with minor modifications is all that is required [8,10]. Such attitudes are damaging to all and neglectful of potential solutions within our grasp [11,12].

The deliberate structuring of the global economy for the preferential benefit of a small proportion of people reflects inadequate regard for the impact of entrenched poverty on the neglected majority [13]. The ongoing extraction of material and human resources from poor countries through interest on debt that can never be repaid, exploitation of land and mineral resources impoverish already poor countries and add severe ecological damage [9,14]. The resulting severely adverse living conditions of many, that foster poor health and allow for only rudimentary health and social services, have been conducive to continuing cross-species transmission of infections like HIV, SARS, Avian ‘flu and Ebola and the persistence and spread of tuberculosis and malaria [2-4]. All of these diseases and the emergence of drug resistance cannot be controlled through biomedical means alone. From a historical perspective, the profoundly disruptive relationships between Africa and the world are additional relevant upstream causal factors, [2,15] and if the Ebola epidemic cannot be contained, its impact, like that of HIV/AIDS, will have a devastating effect on sub-Saharan Africa.

While we seemingly value freedom, human rights, progress based on reason, human dignity, democracy, competitiveness in free markets and economic growth, selective strategies and power relations have distorted the application of these concepts [16-18]. Notable contributions to these distortions and our unstable predicament include an almost unwavering belief in flawed economic dogma (until very recently), an extravagant sense of entitlement to endless consumption and acceptance of the ‘justice of deserved privilege.’

Widespread belief that ongoing biomedical advances will provide all the solutions reflects denial of the magnitude of these global crises, their multifactorial causality, and the covert complicity of the most privileged and powerful in the complex causal chain [19]. Indeed such belief can be seen to be unwarranted by the fact that the benefits of progress and economic growth have largely impacted on the lives of only one third of the world’s population and are not achievable or sustainable for a majority in the context of population growth, wasteful use of limited natural resources and climate change [8,20]. Such features of the global economic landscape are also apparent in many local environments [2,21].

The beneficence of development aid, which is necessary but insufficient, does not adequately reach the intended sources or contribute to ‘development’ when so much is deflected towards urgent humanitarian issues [22]. High profile praise for helpful philanthropy also obscures the facts that extraction of resources from the ‘global south’, including massive repayments on debt that can never be eradicated, results in the net global financial flows from the ‘global south’ to the ‘global north’ far exceeding donor aid [23]. Ineffective local and global governance are also deeply implicated in sustaining poverty, together with overt and covert forms of corruption and exploitation, and other disruptive forces [24].

It is also notable that most publications on international and global health challenges come from those in the ‘global north,’ whose proposed solutions relegate to a lower priority the perspectives from the ‘global south’ [25].

## Conclusions

Failure to respond to the adverse power relations that sustain local and global inequalities is not due to lack of insight, ingenuity or resources. The explanation lies in lack of imagination and a poorly developed sense of local and global social, economic, physical and moral interdependence in the face of ongoing natural, biological and human induced tragedies.

Deeper understanding of the fragility of all lives, including those of privileged and powerful people, couple to wise cosmopolitan political, moral and humanitarian leadership [26,27] could ameliorate looming environmental and social tragedies, that are visible writings on the wall, and promote a trajectory towards a more peaceful and sustainable future [9,28].
